# How to Cope With Stress in the Desert—The Date Palm Approach

**DOI:** 10.1111/pce.15188

**Published:** 2024-10-01

**Authors:** Baoguo Du, Bastian Leander Franzisky, Waqas Muhammad, Saleh Alfarraj, Christoph‐Martin Geilfus, Heinz Rennenberg

**Affiliations:** ^1^ College of Life Science and Biotechnology, Ecological Security and Protection Key Laboratory of Sichuan Province Mianyang Normal University Mianyang China; ^2^ Chair of Tree Physiology, Institute of Forest Sciences University of Freiburg Freiburg Germany; ^3^ Department of Soil Science and Plant Nutrition Hochschule Geisenheim University Geisenheim Germany; ^4^ Department of Zoology King Saud University Riyadh Saudi Arabia; ^5^ Center of Molecular Ecophysiology (CMEP), College of Resources and Environment Southwest University Chongqing China

**Keywords:** antioxidants, climate change, drought, heat, local and systematic responses, ozone, phenolic compounds, salinity, secondary metabolites

## Abstract

Increasing desertification constitutes a global environmental problem, mainly driven by climate change and inappropriate land‐use that limits agriculture, forestry and human colonization. The selection of suitable plant species to mitigate desertification is particularly challenging, as it usually requires simultaneous counteraction against a whole set of unfavourable environmental conditions, including heat, drought, high tropospheric ozone and salinity. It therefore seems useful to identify the survival strategies of plants native in desert environments. Date palm constitutes a plant species native in desert environments and cultivated worldwide in arid regions that have been studied intensively for stress defence during the last decade. The present review summarizes the current state of biochemical stress defence mechanisms including avoidance, osmotic and metabolic adjustments and reactive oxygen species scavenging, addresses whole‐plant regulations and trade‐off between stress compensation/defence and growth of date palms. The review advances our knowledge about how this typical desert species copes with both individual and multiple environmental stresses at the cellular to the whole‐plant level, and identifies areas of future research required to fully understand the strategies of this plant species to survive in the desert, thereby contributing to efforts for the mitigation of climate change and desertification.

## Summary statement

This review summarizes the current state of biochemical stress defence mechanisms with date palms and identifies areas of future research required to fully understand the strategies of this plant species to survive in the desert, thereby contributing to mitigate climate change and desertification.

## 1 Introduction

Date palm (*Phoenix dactylifera* L.) as one of the oldest perennial fruit trees, autochtoneous in desert environments, has been widely cultivated in the Middle East, South Asia, North Africa and Central America due to its socio‐economical importance (Chao and Krueger 2007; Hussain, Farooq, and Syed 2020). Additional cultivation areas have become feasible in West Asia, North and South America as a consequence of global warming (Shabani, Kumar, and Taylor Kumar, and Taylor 2012, 2015). In these arid regions, date palm is facing great challenges from a variety of stresses, for instance from more frequent and serious heat and drought extremes, salinity resulted from irrigation with brackish water and directly from sea water flooding in coastal areas (Hazzouri et al. 2020), as well as steadily increased tropospheric ozone concentration. The latter is formed either locally in these arid and semi‐arid regions from anthropogenic emissions or/and imported by long distance transport of polluted air, for example, from Europe (Lal et al. 2012; Osipov et al. 2022). These constrains, particularly shortage of water, extreme air temperature, soil salinity as well as land degradation and desertification are projected to be intensified in the context of climate change (Ajjur and Al‐Ghamdi 2021; Zittis et al. 2022) and by inappropriate land‐use practices (Huang et al. 2020; Choukr‐Allah et al. 2023). Knowledge of how date palm trees cope with the hassle conditions is of particularly importance to gain a better understanding of defence mechanisms of plants to sole or multiple stresses, and will provide information on the selection of plant species and cultivars suitable for counteracting desertification and its spreading (Stavi, Xu, and Argaman 2024).

**Figure 1:**
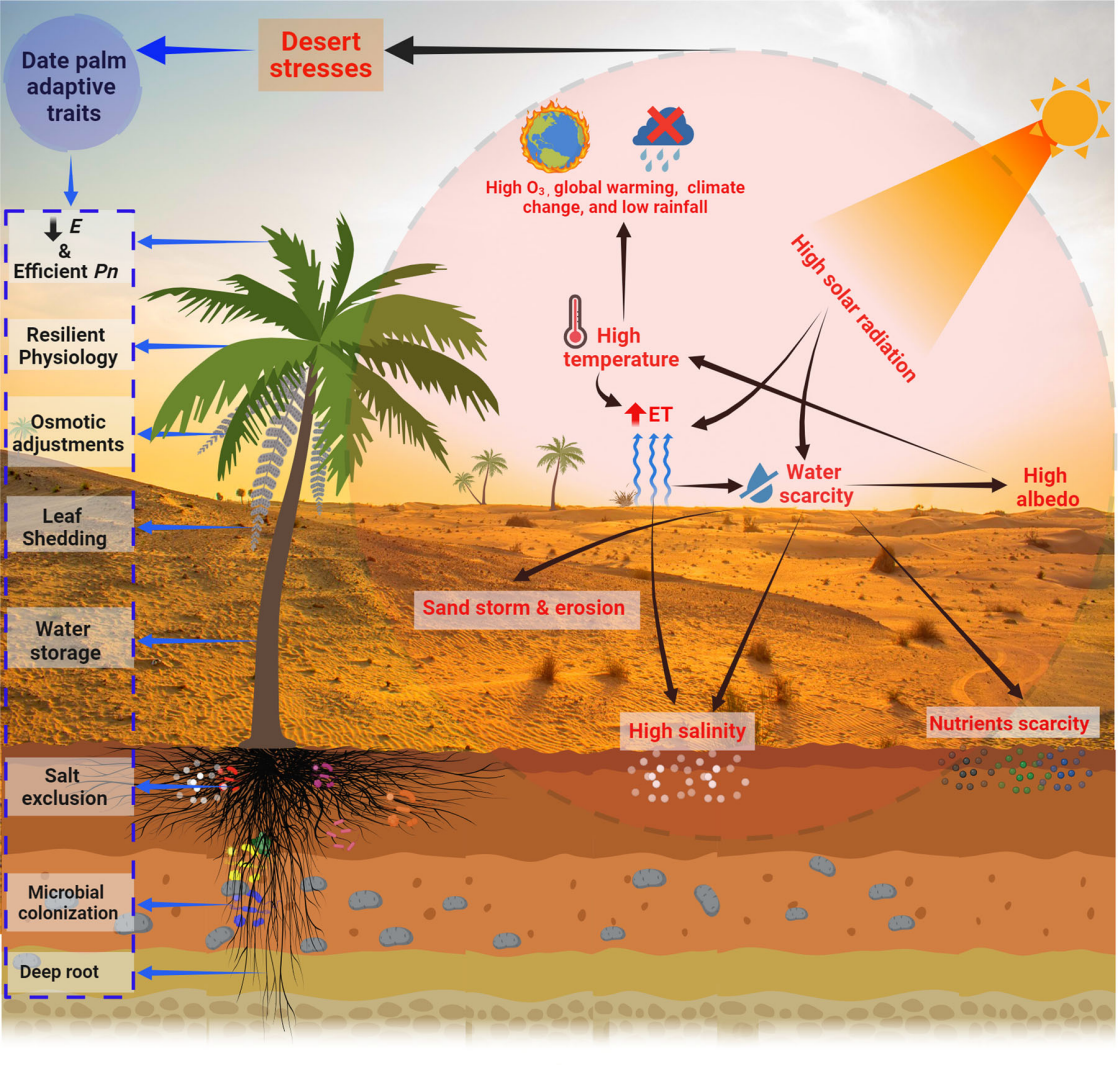
The adverse conditions of the desert, including higher temperatures, high evapotranspiration, high albedo (reflection of solar radiation), ozone level, water and nutrient scarcity, and salinity, pose challenges for desert flora, exacerbating desertification and contributing to global warming and climate change. However, the date palm has the potential to cope with these challenges and reduce the intensification of desertification while mitigating climate change. Date palm traits to accommodate these stresses include the deep root system, microbial colonization, uptake of K^+^ and compartmentation and exclusion of toxic ions (Na^+^, Cl^−^), accumulation of osmolytes, water storage in the trunk, waxy cuticle covering on leaf surfaces, leaf pattern to reduce direct exposure to sunlight, shedding of leaves, activation of heat shock proteins, osmotic adjustments, exceptionally resilient physiology, lowering of transpiration and efficient photosynthesis.

Apart from morpho‐anatomical characteristics such as thick leaves, epidermis, and cuticle, bast fibres around the stem, strong root systems with well^−^developed aerenchyma and intensive sclerification in the vascular region (Fatima et al. 2014) and stomatal regulation, a whole set of complex biochemical processes is involved in stress defence and tolerance, including osmotic adjustment, reactive oxygen species (ROS) scavenging, toxic ion compartmentation and exclusion, as well as metabolic regulations, in a local and systemic manner (Figure 1) (Du et al. 2024). During the last few years a significant body of new studies characterized biochemical mechanisms responsible for the high tolerance of date palms to stresses experienced in the desert. Except for a report by Hazzouri et al. (2020), who reviewed the tolerance mechanisms of date palm to salinity and drought with the focusing on perspectives from omics, microbiomes and future genetic engineering, an intensive review summarizing recent progresses in this area of research is not available. The present review will summarize recent findings, thereby identifying the mechanisms determining the tolerance of date palms to different stresses. In addition, knowledge gaps on the delicate balance of the trade‐off between stress compensation/defence and growth as well as fruit production, energy generation to support defence processes as well as approaches to avoid ion toxicity in the cytosol and other compartments are addressed to shed light on future research avenues.

## 2 Responses to Heat

In their natural environment, date palms frequently experience summer temperatures above 45°C and a diurnal temperature range spanning more than 30°C (Babaousmail et al. 2022). Under these conditions, growth and development of most plant species is impaired, even at sufficient water supply. This is because respiration increases exponentially with increasing temperature, whereas photosynthesis increases only up to a species specific maximum, after which it declines (Rennenberg et al. 2006). Thus, at high temperatures, CO_2_ loss by respiration can exceed CO_2_ fixation by photosynthesis. In date palms, however, this effect is counteracted by a high temperature optimum of photosynthesis ranging from 20°C to 33°C in winter and from 28°C to 45°C in summer climate (Kruse et al. 2019). Current studies suggest date palms optimize photosynthesis and respiration to sustain high energy production under heat stress, which in turn energizes the transcription of heat shock proteins and chaperones, safeguarding metabolic processes. Apparently, acclimation of photosynthesis to high temperatures reflects a balance between maximizing photosynthesis and minimizing the risk of metabolic perturbation (i.e. disruption of enzyme functions) (Kruse et al. 2019). This acclimation response seems to be achieved by enhanced transcription of chaperone proteins including heat shock proteins, and heat stress transcription factors, for example, PDACT_KE332562.1_G003770 and PDACT_KE332562.1_G003780 (Safronov et al. 2017; Aziz et al. 2023), and by adjusting the abundance of proteins of the photosynthetic machinery (Ghirardo et al. 2021). In addition, enhanced expression of two negative, and reduced expression of one positive regulators of cell death was observed. Metabolome analyses revealed a switch to carbohydrate metabolism and cell wall biogenesis in response to heat (Safronov et al. 2017). The role of these metabolic changes in the defence of heat stress remains to be elucidated.

Heat mediated water loss from date palm leaves by cuticular transpiration seems to be strongly reduced by a particular wax and cutin composition. Minimum leaf conductance of date palms was six times lower than for a water‐spender desert plant and did not change in the temperature range of 25°C–50°C (Bueno et al. 2019). We propose that date palms adjust their metabolism to prioritize carbohydrphosphoenolpyruvate carboxylaseate processing and cell wall biogenesis, ensuring energy availability during heat stress. Proline accumulation probably serves dual roles as an osmo‐protectant and antioxidant, stabilizing energy balance (Yaish 2015). The distinctive leaf structure of date palms minimizes water loss, while proline aids in maintaining cellular hydration, thus conserving energy.

Acclimation of date palms to heat also includes optimization of other components of the antioxidative system for ROS scavenging. Date palms likely engage antioxidant systems like glutathione reductase (GR) to manage ROS, thereby preventing oxidative damage and conserving energy for acclimation. This acclimation is indicated by a drop in the level of the anti‐oxidants glutathione and ascorbate accompanied by increased activity of GR. This approach is similar to strategies seen in salt‐tolerant crops, where antioxidant enzymes enhance energy efficiency by reducing ROS production (Munns et al. 2020). Apparently, these changes in the antioxidative system mediate homoeostasis of the redox state, as indicated by unchanged H_2_O_2_ levels at heat exposure. In addition, changes in fatty acid composition seem to contribute to the stabilization of membranes. High isoprene production and emission rates may act as additional scavenger of ROS, thereby further stabilizing thylakoid membranes (Arab et al. 2016). Apparently, enhanced isoprene production and emission is achieved by upregulating the abundance of isoprene synthase (Ghirardo et al. 2021). However, also other components of the antioxidative system including phenolic compounds (Arab et al. 2020), so far not analyzed in date palms, may contribute to the acclimation to heat of this plant species. This view is supported by enhanced expression of numerous genes related to ROS scavenging in cytosol, chloroplasts and peroxisomes (Safronov et al. 2017).

In addition, genes differentially expressed in response to heat were enriched for circadian and diurnal rhythm motifs (Safronov et al. 2017). This observation indicates acclimation to the high diurnal temperature range experienced by date palms in their natural environment. The mechanisms involved in this acclimation need attention by future research. Compared to shoots, information about the responses of roots to elevated air and, consequently, soil temperature and stress signalling from shoot‐to‐root are limited and, therefore, deserve future research efforts, which is of particular importance for plants like date palm in arid regions with sandy soil (Tiwari et al. 2022).

## 3 Responses to Drought

Drought is a major limiting factor for plant growth and development, originating from limited precipitation, high evaporation, and human activities like groundwater overuse, but is most severe in desert environments. However, the inherent xerophilic (drought‐tolerant) nature of date palms makes them well adapted to desert ecosystems. Nevertheless, extreme dryness poses significant challenges for date palm growth and yield. For instance, in semi to hyperarid environments, such as the Arabian Peninsula, soil water deficit represents a prevailing challenge for date palm growth and productivity (Yaish and Kumar 2015; Allbed, Kumar, and Shabani 2017). To cope with the desert environment, date palm shows anatomical adaptions (Hazzouri et al. 2020). By germinating below the soil surface, meristems and organs are protected from heat and dry soil by remote germination (Xiao et al. 2019). This is referred to delayed emergence of the seedlings. Moreover, the date palm develops a complex root system that includes laterally extending main and anchor roots, as well as negatively geotropic roots known as pneumatophores, which grow above ground. The suberized outer layers and fibre cells of the main root and the pneumatophores growing near the soil surface represent structurally adaptations that minimize water loss and aid water uptake during sporadic rainfalls, respectively (Xiao et al. 2019). In addition, date palm develops sclerotic leaves with relatively low transpiration and CO_2_ assimilation rates, and grows relatively slowly even at well water supply (Kruse et al. 2019). Leaves exhibit stomata of the tetracytic type surrounded by epicuticular wax chimneys that increase the boundary resistance for stomatal transpiration (Müller et al. 2017). However, depending on the extent and duration of soil desiccation, leaf hydration can be significantly reduced by up to 80% (Du, Kruse, et al. 2021; Alhajhoj et al. 2022; Du et al. 2023).

In response to soil desiccation, date palm roots and leaves accumulate the stress hormone abscisic acid (ABA) (Shareef, Abdi, and Fahad 2020; Garcia‐Maquilon et al. 2021) with higher concentrations in the leaves than in roots (Franzisky et al. 2024). In date palm, ABA is sensed by receptors of the PYL8‐like family that accumulate in response to ABA treatment, presumably boosting ABA signalling to induce drought acclimation (Garcia‐Maquilon et al. 2021). ABA‐related stomatal closure is mediated by the ABA kinase *P. dactylifera* OST1 that activates the *P. dactylifera* SLAC1‐type anion channel at the guard cell plasma membrane (Müller et al. 2017). Notably, nitrate availability at the extracellular domain of *P. dactylifera* SLAC1 accelerates ABA‐mediated stomatal closure, suggesting a role of nitrate as signalling molecule linking the nitrogen status of the plant to the performance of the stomata. Stomatal closure limits transpirational water loss along with the adjustment of hydraulic conductivity, as indicated by drought‐related reduction of aquaporin abundance (Patankar, Al^−^Harrasi, Al−Yahyai & Yaish 2019). However, the reduced stomatal conductance, in turn, reduces photosynthetic performance. The reduction varies from moderate (Arab et al. 2016) to severe in response to long‐term drought exposure (Alhajhoj et al. 2022).

Drought leads to chlorophyll degradation (Du et al. 2019; Anli et al. 2020; Du, Kruse, et al. 2021; Akensous et al. 2022; Alhajhoj et al. 2022; Ali‐Dinar, Munir, and Mohammed 2023) and changes in the photosynthetic machinery (El Rabey, Al‐Malki, and Abulnaja 2016; Ghirardo et al. 2021; Alhajhoj et al. 2022), which likely reduce light‐harvesting due to the reduction in photosystem antennae. As a result, the rate of photosynthetic electron transport is reduced, mitigating the risk for over‐reduction of electron acceptor pools and the resulting formation of ROS due to unfavourable temperature and water regimes at continuous sunlight exposure (Kruse et al. 2019). With respect to the stability of thylakoid membranes under drought, a change in membrane lipid composition has been documented. In addition to an increase in unsaturated lipids that might counteract stress‐related increase in membrane fluidity (Arab et al. 2016; Du, Kruse, et al. 2021), an enhanced expression of genes related to plastid lipid import and the replacement of mono‐galacto‐ by di‐galactolipids in thylakoid membranes, relevant for stability under abiotic stress, was observed (Franzisky et al. 2024). Similarly, also isoprene synthesis and emission are upregulated (Arab et al. 2016; Ghirardo et al. 2021), which might further aid in stabilizing thylakoid membranes by preventing lipid peroxidation.

Along with photosynthesis, other metabolic pathways are significantly affected by drought exposure, leading to the accumulation of amino acids and sugars (Safronov et al. 2017; Du, Kruse, et al. 2021; Du et al. 2023; Franzisky et al. 2024). Particularly in date palm roots, the accumulation of oligosaccharides suggests a significant role of organic compounds in osmotic adjustment of roots compared to the moderate increase of mineral osmolytes such as potassium (Franzisky et al. 2024). Also numerous free amino acids, such as the compatible solute proline, accumulate (Yaish 2015; Shareef, Abdi, and Fahad 2020; Du, Kruse, et al. 2021; Franzisky et al. 2024) and might be involved in stabilizing cell structures and scavenging ROS. Already at the beginning of drought, accumulation of oxidized derivatives of ascorbate and glutathione, dehydroascorbate and glutathione disulfide in roots and leaves indicate an increased demand for ROS metabolism (Du, Kruse, et al. 2021; Franzisky et al. 2024). Consequently, the antioxidant machinery is activated in drought exposed date palm. In addition to higher activities of antioxidant enzymes, intermediates of glutathione biosynthesis appear to be increased immediately after the onset of drought (Franzisky et al. 2024). After several weeks of continuous water deficit, coherent changes in gene expression, enzyme abundance and metabolite pools indicate a constant need for ROS detoxification. This is consistent with increased foliar concentrations of H_2_O_2_ (Benhiba et al. 2015; Anli et al. 2020; Shareef, Abdi, and Fahad 2020; Akensous et al. 2022; Franzisky et al. 2024) and markers of lipid peroxidation such as malonaldehyde (Benhiba et al. 2015; Anli et al. 2020; Shareef, Abdi, and Fahad 2020; Akensous et al. 2022) observed under long‐term drought exposure. Apparently, the increased antioxidant activity cannot fully mitigate increased ROS formation in leaves of date palms exposed to continuous drought.

The success of the date palm in dry environments is not due to any singular, extraordinary trait, but originates from a combination of well^−^understood anatomical and physiological features, such as moderated growth and efficient water management (Figure 1). Through activating mechanisms like stomatal control to minimize water loss, it adeptly handles scarce moisture, though faces challenges at sustained drought. Further, the enhanced drought tolerance of Arabidopsis achieved by the expression of date palm genes encoding the aquaporine PIP1;2 (Patankar et al. 2019) and abscisic acid receptors (Garcia‐Maquilon et al. 2021), respectively, suggests an enhanced functional efficacy of date palm proteins with respect to drought acclimation responses. In addition to elucidating the molecular mechanisms underlying these observations, it seems promising to clarify whether these date palm genes can be exploited to improve the drought tolerance in glycophytic crops.

## 4 Responses to Salt

Salinity in desert ecosystems is triggered by a complex mix of elements such as scarce rainfall, elevated rates of evaporation, closeness to coasts where shore winds transport salt particles from sea‐spray, and rising groundwater saltiness due to the intrusion of saltwater into freshwater reservoirs. Human practices, especially faulty irrigation practices, exacerbate these issues (Al‐Muaini et al. 2019; Lassiter 2021). Although date palm has an exceptional ability to withstand saline conditions, several lines of evidence indicate that date palm production is compromised when irrigated with saline water (Ramoliya and Pandey 2003; Tripler, Ben‐Gal, and Shani 2007; Tripler et al. 2011; Alhammadi and Edward 2009; Al‐Qurainy et al. 2017; Hazzouri et al. 2020). Hence, there is urgent need for dedicated research and strategic interventions to sustain and expand date palm cultivation in the face of the rising salinity levels in semi‐ to hyperarid regions such as the Arabian Peninsula and Tunisian Sahara, where higher salt concentrations are proven to be problematic even for this resilient species (Haj‐Amor et al. 2016; Alfarrah and Walraevens 2018).

In saline environments, plant growth is compromised due to several factors. In the initial phase lasting from hours to days, the water uptake of the plant is disrupted due to the increased salt ion concentration in the rooting medium that decreases the water potential of the soil solution. For adjustment, stomatal pores rapidly close (hours) which stabilizes shoot hydration (Munns and Tester 2008). Next, root osmotic adjustment helps to restore water uptake in the long term (days ‐ weeks). However, typically within weeks, after onset of salt exposure, there is an excessive uptake of salt ions along with water from the soil, causing ion toxicities in the cell, further hampering plant growth. Strategies to avoid salt ion accumulation to toxic levels are discussed below. In date palms exposed to salt, the plant stress hormone abscisic acid (ABA) accumulates rapidly in roots and leaves (Mueller et al. 2023), causing stomatal closure (Tripler, Ben‐Gal, and Shani 2007; Müller et al. 2017). In addition to the reduction in stomatal aperture, the sclerotic nature of the leaves with stomata surrounded by waxy chimneys also aids in reducing transpirational water loss (Müller et al. 2017). In response to salt exposure, the CO_2_ assimilation rate of date palm leaves decreases (Sperling et al. 2014; Yaish et al. 2017; Du, Ma, et al. 2021). However, the reduced CO_2_ assimilation is likely not a consequence of damage to the photosynthetic apparatus. Rather, it originates from increased non‐photochemical quenching (Sperling et al. 2014), a protective mechanism that dissipates excess light energy as heat, thus preventing damage to the photosystem. The decrease in light‐harvesting chlorophylls under salt exposure (Al‐Qurainy et al. 2017; Shareef, Abdi, and Fahad 2020; Ait‐El‐Mokhtar et al. 2020) likely also decreases light absorption and electron transport rate, with chlorophylls decreasing less in tolerant cultivars (Al Kharusi, Al Yahyai, and Yaish 2019).

Furthermore, the phosphoenolpyruvate carboxylase (PEPC), which initiates the first carboxylation reaction in C4 and CAM photosynthesis, was unexpectedly upregulated in date palm under salinity stress (Yaish et al. 2017). This finding in a C3 plant suggests a potential induction of C4 or CAM pathways, warranting further investigation. Activation of these pathways is known to be crucial for salt tolerance. Reduced stomatal conductance might cause depletion of leaf internal CO_2_ and negative cellular redox potential leading to increased generation of ROS (Allen 1995; Lawlor and Tezara 2009). In line, already after 1‐day salt exposure, metabolites related to the glutathione biosynthesis pathway increase (Mueller et al. 2023) suggesting an immediate demand of glutathione in the course of H_2_O_2_ detoxification via the Foyer‐Halliwell^−^Asada cycle. This frequently observed pattern is also evident in the long‐term response to salt exposure (Al‐Qurainy et al. 2017; Al Kharusi, Al Yahyai, and Yaish 2019) and is accompanied by increased protein abundance of antioxidant enzymes such as ascorbate peroxidases and glutathione S‐transferases in roots and catalases and superoxide dismutases in leaves (Mueller et al. 2023). High antioxidative capacity is a feature of salt tolerance (Al Kharusi, Al Yahyai, and Yaish 2019) and particularly antioxidant enzymes might be stimulated by application of biofertilizers and microbial inoculum (Naser et al. 2016; Outamamat et al. 2021; Sabeem et al. 2022). In parallel to this upregulation of the antioxidant machinery already after 1‐day salt exposure, date palm rapidly accumulates compatible solutes such as proline (Yaish 2015; Yaish and Kumar 2015; Al Kharusi, Al Yahyai, and Yaish 2019; Al Kharusi et al. 2019) and other amino acids (Xu et al. 2022; Du et al. 2023; Mueller et al. 2023). Proline accumulation, for example, is achieved by increased expression of the gene coding for the rate limiting enzyme of its biosynthesis, delta‐1‐pyrroline‐5‐carboxylate synthase 1 (P5CS), while a catabolic gene appeared downregulated. The extent of proline accumulation is suggested to positively correlate with salt tolerance in date palm (Hazzouri et al. 2020), because cultivars such as Zabat that are considered less salt tolerant, exhibit weaker or no accumulation of proline (Al Kharusi, Al Yahyai, and Yaish 2019). The accumulation of other compatible solutes such as sugars in salt exposed date palm remains ambiguous, with values increasing or remaining unchanged in two varieties considered salt tolerant, i.e., Umsila (Al Kharusi, Al Yahyai, and Yaish 2019) and Khalas (Du et al. 2023; Mueller et al. 2023).

Plants exposed to high NaCl concentrations experience disrupted ionic homoeostasis, leading to stress and potential toxicity. Date palm prevents toxic salt ion accumulation in its photosynthetically active leaves by keeping Na^+^ and Cl^−^ levels low, despite their high presence in the surrounding soil solution (Alhammadi and Edward 2009; Al‐Bahrany and Al‐Khayri 2012; Al Kharusi et al. 2017). Particularly in cultivars classified being tolerant, increases in foliar Na^+^ and Cl^−^ are largely prevented or kept at low levels (Alhammadi and Edward 2009; Sperling et al. 2014; Al Kharusi, Al Yahyai, and Yaish 2019a), highlighting its strong ability to restrict xylem loading in the roots and thus long‐distance transport of these ions that would cause accumulation above unacceptable cellular thresholds. With respect to roots in direct contact with the saline soil solution, the suberinized outer layers and fibre cells of the main root of date palm are considered an anatomical adaptation that prevents apoplastic bypass of ions to the vasculature, thus helping to balance ion fluxes (Xiao et al. 2019). Knowledge of Cl^‐^ selective transporters involved in salt tolerance is yet limited. Therefore, the mechanism by which date palm achieves exclusion, viz. restricts xylem loading of Cl^‐^ remains elusive. Increased abundance of Cl^‐^ transporters involved in Cl^‐^ release, such as SLAH1/4 and SLAH3, have been reported in date palm roots upon salt exposure (Mueller et al. 2023), although their exact cellular localization in the root remains to be determined. Assuming a SLAH1/4 expression in the root cortex, an exclusion function with release of Cl^‐^ into the cell wall would be conceivable. In addition, most of the nitrate transporter NRT orthologous detected (11 out of 13) were downregulated up to 40‐fold in salt‐exposed roots. Considering that other NRT orthologues, similar to NRT1.1 (NPF6.3) in *Arabidopsis thaliana*, have been suggested to be involved in Cl^‐^ uptake in *Medicago truncatula* (Xiao et al. 2021), this prominent reduction of NRTs in salt‐exposed date palm roots could contribute to the reduction of Cl^‐^ uptake at the root level. However, the unexpected upregulation of SLAH3, which is thought to be involved in Cl^‐^ translocation to leaves (Cubero‐Font et al. 2016), and the downregulation of NPF2.5 and NAXT1, which are thought to be responsible for Cl^‐^ release into the soil ((Teakle and Tyerman 2010) and references therein), highlight the need for further research on function and localization of Cl^‐^ transporters to understand the regulation of Cl^‐^ hoemostasis in salt‐exposed date palm roots.

To achieve ionic homoeostasis, we hypothesize that date palms use specific transporters, such as Na^+^/H^+^ antiporters and Ca^2+^‐ATPases, to compartmentalize salts within vacuoles, protecting cytosolic processes. This reduces their harmful presence in the cytosol and stabilizes cellular structures, as described elsewhere (Niu et al. 1995). The mechanism for excluding Na^+^ is better understood compared to Cl^‐^. The expression of date palm *SOS1* is strongly induced in roots (Xu et al. 2022), particularly in sections with high Na^+^ concentrations, i.e., the few suberinized root tips where unselective apoplastic ion entering is likely (Mueller et al. 2023). Additionally, date palms may activate pathways to expel excess sodium and produce osmo‐protectants like proline, balancing osmotic pressure within cells. This strategy mirrors the way how cotton is maintaining ionic balance and enzyme activity under salt stress (Guo et al. 2020). SOS1 is an orthologue of the *Arabidopsis thaliana* plasma membrane‐localized Na^+^/proton (H^+^) antiporter of the NHX (Na^+^/H^+^ exchanger)‐family. It facilitates Na^+^ release from cytosol into the apoplast or soil, thereby regulating cytosolic Na^+^ concentration and hence ensuring that cytosolic enzymes remain functional under high salinity conditions as observed in cotton (Guo et al. 2020). In addition to upregulation of SOS1 to enhance Na^+^ efflux, potential Na^+^ uptake pathways are reduced by a strong downregulation (Mueller et al. 2023) of numerous orthologues of the Na^+^‐permeable ion channels of the HKT family (Uozumi et al. 2000; Zhang, Flowers, and Wang 2010; Waters, Gilliham, and Hrmova 2013). These root transportome adjustments allow date palm to reduce the uptake of Na^+^ intake (Radwan et al. 2015; Yaish et al. 2017; Mueller et al. 2023) by restricting uptake and maximizing efflux. In addition, the expression of SOS1, among other stress‐related genes, has been suggested to interact with the presence of microbes. Root colonization by the endophytic fungus *Piriformospora indica* led to increased *SOS1* expression. Consequently, fewer Na^+^ per K^+^ accumulated in roots and leaves (Sabeem et al. 2022). Silicon application also had a similar effect. However, this was more likely due to the strengthening of the Casparian strip barriers in the epidermis and endodermis (Jana et al. 2019), as previously observed in the relatively salt‐tolerant date palm cultivar Umsila, but not in the less tolerant Zabad (Al Kharusi, Al Yahyai, and Yaish 2019). Thicker Casparian strips limit nonselective ion entry into the root stele via the apoplastic pathway and, thus, nonselective bypass of Na^+^ under salinity. Therefore, the remarkable inherent ability of date palm to exclude mineral ions may be further enhanced by, for example, microbiota inoculation and silicon application (Hazzouri et al. 2020).

Taken together, date palm thrives in salt‐affected terrains due to a synergistic assembly of mechanisms such as efficient salt sequestration and ion balance optimization, rapid adjustment of transpiration along with photosynthetic and antioxidative metabolism (Figure 1). These strategies, common among various plants are refined for harsh, dry environments, and drive its productivity. This approach underscores the significance of applying known resilience traits in agriculture to enhance yield in areas facing salinity challenges.

## 5 Responses to Acute and Chronic Ozone Exposure

Increased levels of tropospheric ozone (O_3_) have been recognized as one of the most significant air pollution negatively affecting growth and development of many plant species (Agathokleous et al. 2020; Nowroz et al. 2024). Of note, date palm in the Middle East and particularly the Arabian Gulf region are subjected to high tropospheric ozone pollution (Smoydzin, Fnais, and Lelieveld 2012). However, up‐to‐date only few studies were conducted about the responses of date palm to O_3_. As the first target of tropospheric ozone exposure, reduction stomatal aperture to prevent high O_3_ uptake is the common response of many plant species (Agathokleous et al. 2020). Acute ozone exposure had little effects on stomatal conductance (200 ppb for 8 h) (Du et al. 2018), but significantly declined stomatal opening under chronic ozone exposure (_3_^−^month exposure at 1.5 and 2 times of control at 45 ppb), particularly in the morning (Paoletti et al. 2021; Hoshika et al. 2024). This effect may be due to stomatal sluggishness caused by high ozone levels, and a strong control of stomatal sensitivity by atmospheric water pressure deficit (Paoletti 2005; Hoshika et al. 2019; Hoshika et al. 2020; Paoletti et al. 2021). Chronic exposure to ozone results in limited ozone uptake by plants, which negatively impacts photosynthesis. This decrease in photosynthesis occurs because the plants gain less carbon, together with a decline in pigment content and the activity of photosystem II (PS II) (Cotrozzi et al. 2020; Paoletti et al. 2021; Hoshika et al. 2024), and foliar contents of amino acids due to reduced availability of carbohydrate in the tricarboxylic acid cycle (TCA cycle) which serve as precursor for the amino acids (Du et al. 2018; Arab et al. 2022). Under elevated ozone exposure, date palm is able to conserve steady soluble sugar concentrations in the leaves (Du et al. 2018; Arab et al. 2022), but the decreased foliar protein contents, enzymes activities and cell wall lignification under longer‐term ozone exposure warrants further study (Arab et al. 2022). Under high ozone exposure, leaf ROS levels of date palm were well controlled most likely by stimulated antioxidant levels including ascorbic acid (ASA), phenolic compounds and flavonoids, either through enhanced *de novo* biosynthesis (ASA, particularly under higher temperature and ozone concentration) or reduced turnover at reduced production (phenolic compounds and flavonoids) (Du et al. 2018; Arab et al. 2022). In addition, changes in the fatty acid composition and elevated emission of monoterpenes (mainly aldehyde volatiles of octanal and nonanal) may also have contributed to the ozone tolerance of date palm (Du et al. 2018; Paoletti et al. 2021).

Next to biochemical responses in leaves, also characteristic traits of date palm roots were strongly impacted by ozone exposure. This observation can be attributed either to activated systematic responses through long‐distance communication or to direct effects of O_3_ penetrated the soil as well as its long‐distance transport inside the plant. Apparently, root uptake and long‐distance translocation of anions (NO_3_^‐^, PO_4_^3−^, SO_4_^2−^) (Du et al. 2018) and cations (Ca, Mg, Fe, Zn, Na and K) (Arab et al. 2023) are impaired under elevated O_3_. The significant decline of foliar NO_3_^−^ concurrent with increased Cl^−^ contents may have contributed to keep turgor and electrical charge balance and guard cells movement, hence, to the high tolerance of date palm seedlings to elevated ozone (Hänsch and Mendel 2009; Vainonen and Kangasjärvi 2015). The significant decline of root biomass under chronic elevated O_3_ exposure (Paoletti et al. 2021) may partially explain the reduced mineral ion uptake. Phloem transport was largely maintained under acute O_3_ exposure as seen from the ozone‐independent partitioning profiles between shoots and roots, including the majority of carbohydrates, ascorbate and amino acids (Herschbach, Scheerer, and Rennenberg 2010; Du et al. 2018). Similar results were observed in other ozone‐resistant species, e.g., loblolly pine and wheat (Spence, Rykiel, and Sharpe 1990; Mortensen and Engvild 1995). To compensate reduced foliar carbon metabolism under chronic elevated O_3_ exposure, date palm reduced phloem loading and allocation of carbohydrate from the leaves to the roots, as evidenced by the significantly reduced transcript abundance of a root sucrose transporter (PdSUT3) (Arab et al. 2022), slowed down root carbon metabolism (Arab et al. 2022) and, consequently, hindered root growth (Paoletti et al. 2021). However, only little information is available about the effects of O_3_ exposure on the activity of carbohydrate transport proteins. Therefore, further studies are required to identify and characterize sugar transport processes at ozone exposure and the regulatory mechanisms involved.

Apparently, roots experienced more oxidative pressure than leaves upon ozone exposure as indicated by the increased ROS and lipid peroxidation levels as well as insufficient regulation of ROS levels by the Foyer‐Halliwell Asada cycle (Arab et al. 2022). Phenolic compounds were accumulated in response to acute O_3_ exposure (Du et al. 2018), but decreased together with flavonoids under chronic O_3_ exposure due to carbohydrate deficiency (Arab et al. 2022). This latter view was supported by severely declined concentrations of numerous sugars and organic acids, as well as activities of central enzymes, i.e., phenylalanine ammonia‐lyase, shikimate dehydrogenase and cinnamyl alcohol dehydrogenase involved in secondary compounds synthesis (Arab et al. 2022).

Collectively, apart from ozone tolerance leaf anatomical features such as thick cuticle and wax layer and high stomatal resistance (Khelil et al. 2016), the limited studies reported indicate high ozone resistance of date palm seedlings through well‐tuned local and systemic changes in both, primary and secondary metabolisms, carbon assimilation, roots uptake and growth that were supressed under chronic O_3_ exposure. Since the effects of O_3_ exposure are dependent on the dose of cumulative O_3_ uptake (CLRTAP 2017), additional long term studies with relative high ozone concentrations are urgently required.

## 6 Comparison of Individual, and Analyses of Multiple Stress Responses

Only few studies compared the responses of date palm to different types of stress or analysed multiple stress responses (Table 1). However, for the evaluation of similarities and differences between responses to different types of stress, comparison in the same study is required, because different studies mostly used different experimental approaches. In this context, it also has to be considered that for some stresses, such as salinity, flooding and root zone drying, roots are the first target and stress responses of the shoot are the consequence of root‐to‐shoot communication. For other stresses, such as heat and ozone exposure, shoots are the first target and stress responses of the root are the consequence of shoot‐to‐root communication.

**Table 1:** Summary of various mechanisms in the date palm to adapt to desert conditions. ✓ indicates occurrence, ✗ indicates absence, ii implies indirect involvement, ‘un’ denotes unknown.

Mechanism	Heat	Drought	Salt	Ozone
Unique wax and cutin	✓	✓	✓	✓
ROS scavenging metaboilsm	✓	✓	✓	✓
Sclerophyllous leaves	✓	✓	✓	✓
Antioxidant defence	✓	✓	✓	✓
Upregulation of signalling molecules	✓	✓	✓	✓
Ionic adjustments	✓	✓	✓	✓
Osmoprotection	✓	✓	✓	✓
Stomatal regulation	✓	✓	✓	✓
Limiting carbon assimilation	✓	✓	✓	✓
Epicuticular wax chimneys	✓	✓	✓	ii
Diurnal temperature acclimation	✓	✗	✗	✗
Microbial defence	✓	✓	✓	✓
Deeper root	✓	✓	✓	✗
Heat shock proteins upregulation	✓	✓	✓	un
Suberized layers and fibre cells	✓	✓	✓	✓
Increase Na+ and Cl‐ efflux, decrease influx	✗	✗	✓	✗
Isoprene production	✓	✓	✓	un
Improve WUE	✓	✓	✓	ii
Upregulation of PEPC	un	un	✓	un

### 6.1 Comparison of Individual Stress Responses

The few studies that compared responses of date palm to individual stresses under the same experimental conditions were mostly focused either on the comparison of drought and salinity (Patankar et al. 2019; Du et al. 2023; Mueller et al. 2023; Franzisky et al. 2024) or on the comparison of heat and drought (Arab et al. 2016; Safronov et al. 2017; Kruse et al. 2019; Ghirardo et al. 2021). Flooding or ozone exposure were not included in such studies. Only one study reported the comparison of heat, cold, drought and salinity, but with a restricted data‐set (Yaish 2015).

#### 6.1.1 Comparison of Drought and Salinity Responses (First Target of Both: The Roots)

In general, strategies of plants to acclimate to both salinity and drought show both, differences and similarities (Du et al. 2023, 2024; Mueller et al. 2023; Franzisky et al. 2024). To acclimate to salinity, avoidance of Na uptake by the roots and maintenance of Na/K homoeostasis constitutes a common strategy. Both, salinity and drought require osmotic adjustment by the synthesis of compatible solutes, but also stomatal closure, in the case of salinity to reduce Na and Cl allocation from the roots to the leaves, in the case of drought to reduce water loss. In both cases, stomatal closure is accompanied by enhanced ROS generation that is defeated by accelerating components of the antioxidative metabolism.

In date palm, avoidance strategies play an important role for coping with salinity and drought. Upon salt exposure, excess root uptake of Na is avoided by reduced transporter activity and Na allocation from the roots to the shoots is diminished by stomatal closure (Mueller et al. 2023). In response to drought, stomatal closure prevents excessive water less, i.e., wilting (Franzisky et al. 2024). The high significance of stomatal closure in preventing salinity and drought stress is also indicated by heterologous expression of the date palm aquaporin *PdPIP1;2* in *A. thaliana* (Patankar et al. 2019). This aquaporin is accumulated in date palm upon drought and increases biomass, chlorophyll content, root length, and K/Na ratios under both drought and salinity in transgenic *A. thaliana*.

Other defence strategies to salinity and drought of date palm are realized differently in roots and leaves. Both drought and salinity reduce total C and enhance total N content in the roots, but only salinity enhances total C in the leaves at prolonged exposure (Du et al. 2023). This result indicates uncoupling of C and N metabolism under both stress conditions. In response to soil desiccation, the osmotic strength of date palm roots, but not of leaves increases. Consequently, osmotic adjustment by accumulation of organic osmolytes such as oligosaccharide and amino acid accumulation is recorded in the roots, but not in the leaves (Du et al. 2023; Franzisky et al. 2024). In response to salinity, osmotic adjustment by organic osmolytes is observed not only in the roots but also by amino acid accumulation in the leaves (Du et al. 2023). Accumulation of the amino acid proline in date palm roots and leaves has also been recorded in a study that compared responses to salinity, drought, cold and heat exposure. The authors recorded proline accumulation at all stresses applied, but particularly high proline levels in the cold and salinity treatments (Yaish 2015). Also, stimulation of ROS scavenging, required anti‐oxidants as a consequence of enhanced ROS production due to stomatal closure, differs between drought and salt exposure. Upon salinity, stimulation of ROS scavenging by anti‐oxidants and the Foyer‐Haliwell‐Asada cycle is observed predominantly in the leaves, upon drought in both, roots and leaves (Mueller et al. 2023; Franzisky et al. 2024).

#### 6.1.2 Comparison of Heat (First Target: The Leaves) and Drought (First Target: The Roots) Responses

Stomatal closure is a common response of plants to both, heat and drought (Rennenberg et al. 2006), though with different aims. Upon drought, stomatal closure will prevent excessive water loss from the leaves by transpiration, however, at the expense of reduced photosynthetic CO_2_ fixation; upon heat, stomatal closure reduces the respiratory CO_2_ loss from the leaves, when photosynthesis declines at increasing temperature, but respiration further increases to meet the demand for energy to support cellular repair, along with the acceleration of biochemical reactions (Kruse et al. 2019). However, this reduction in photosynthesis is accompanied by decreased cooling of the leaf surface by transpiration (Blasini et al. 2022).

In date palm, comparison of responses to heat and drought exposure revealed differences between a short‐term growth chamber experiment (Arab et al. 2016; Safronov et al. 2017) and simulated summer and winter climate in a phytotron experiment (Kruse et al. 2019; Du, Kruse, et al. 2021; Ghirardo et al. 2021). In the growth chamber experiment, heat and water deprivation enhanced photosynthetic CO_2_ fixation and stomatal conductance, but reduced the internal CO_2_ concentration. Isoprene emission increased only in response to heat, probably for the stabilisation of thylakoid membranes. Fatty acid composition of the leaves only reacted to drought, indicating that fatty acid composition was constitutively adapted to heat (Arab et al. 2016). Both treatments showed similar transcriptional activation of genes related to ROS scavenging (Safronov et al. 2017), but distinct differences in metabolic features of ROS metabolism (Arab et al. 2016). In leaves, heat reduced antioxidant contents were overcompensated by enhanced GR activity with the result of stable H_2_O_2_ contents, whereas effects of drought on antioxidant contents and GR activity were not observed. In roots, antioxidant contents were not affected by heat, but partially enhanced by drought, whereas GR activity decreased in response to drought, but increased in response to heat. Apparently antioxidative metabolism is differently regulated in leaves and roots in response to heat and drought. Comparison of foliar traits of date palm grown in simulated winter and summer climate (heat exposure) in the phytotron study revealed only small effects on photosynthetic CO_2_ fixation, but enhanced isoprene emission and reduced antioxidant contents as also observed in the growth chamber experiment. Water deprivation enhanced the foliar GSH and GSSG level as well as the total amino acid contents in simulated winter climate upon drought exposure in the phytotron, but not in the growth chamber study (Du, Kruse, et al. 2021; Ghirardo et al. 2021). These results indicate adaptation of date palm to seasonal changes in their environment that can be identified only partially in short‐term growth chamber experiments.

### 6.2 Analyses of Multiple Stress Responses

In their natural environment date palms are usually exposed to a combination of different stresses rather than individual environmental constrains. However, responses of date palm to multiple stresses were only rarely studied. These studies include heat plus drought (Arab et al. 2016; Safronov et al. 2017; Kruse et al. 2019; Du, Kruse, et al. 2021; Ghirardo et al. 2021) and salinity plus flooding (Du, Ma, et al. 2021) under controlled conditions. In a field experiment, the responses of date palm leaves to both, salinity and drought were analysed in combination with heat in summer climate (Shareef, Abdi, and Fahad 2020).

#### 6.2.1 Heat Plus Drought Under Controlled Environmental Conditions

The response of date palm to combined treatments with heat and drought depended on stress intensity. At a mild stress exposure, transcriptional and physiological changes largely resembled those observed for individual heat exposure (Arab et al. 2016; Safronov et al. 2017). These changes included enhanced leaf hydration despite elevated stomatal conductance as well as increased photosynthetic CO_2_ fixation resulting in decreased intercellular CO_2_ concentration. In this mild combined heat and drought treatment, enhanced GR and DHAR gene expression and activity in leaves and roots mediated stable H_2_O_2_ levels, despite decreased contents of ascorbate (AsA) and GSH as well as GSH precursors in the leaves. In roots, GSH levels and its precursors were increased, but AsA levels were unaffected by the combined treatment. In the metabolite data of the mild treatment, combined heat and drought largely resembled the individual drought treatment, indicated by enhanced carbohydrate and amino acid metabolism including proline accumulation (Safronov et al. 2017). At severe drought in simulated summer climate, protein analyses revealed upregulation of the abundance of heat shock proteins and the antioxidative system, but downregulation of photosynthesis and secondary metabolism except for isoprene synthase (Kruse et al. 2019; Ghirardo et al. 2021). Under these conditions, leaf hydration and the concentrations of AsA, most sugars, phenolic compounds, and primary and secondary organic acids were decreased, while thiol, amino acid, raffinose and individual fatty acid contents were increased (Du, Kruse, et al. 2021). Despite enhanced isoprene synthase abundance, emission of VOCs including isoprene were decreased. These results indicate that a metabolic network including the antioxidative system, osmotic adjustment, maintenance of membrane stability, as well as enhanced chaperone abundance are required in date palm to counteract the combine effects of heat and severe drought.

#### 6.2.2 Salinity Plus Flooding Under Controlled Conditions

The natural environment of date palm is not restricted to central parts of the desert, but rather extents to the coast line of the Arabian golf, where the plants are frequently exposed to flooding with sea water. This exposure constitutes two different types of stresses, i.e., salinity and anaerobic conditions in the rhizosphere. Therefore, the combined effect of salinity and flooding was analysed in date palm by experimental sea water expose (Du, Ma, et al. 2021). This treatment reduced CO_2_ assimilation, transpiration and stomatal conductance, but did not affect isoprene emission. The reduced transpiration may partially prevent Na and Cl allocation from the roots to the shoot. Sea water flooding also reduced the abundance of compatible solutes, such as sugars and sugar alcohols, in leaves, but mediated accumulation of compatible N solutes in the roots. Similar responses were not observed by flooding with tap water. Thus, the responses of date palm to sea water flooding largely resembled exposure to salinity. They indicate that date palms are somewhat tolerant to sea water, but highly tolerant to flooding.

#### 6.2.3 Salinity or Drought, Both Plus Heat Under Field Conditions

In a field study, leaves on 3‐ to 4‐year‐old shoots of adult date palms were exposed to drought or salinity in summer (July, August: app. 45°C) or autumn (September: app. 35°C) (Shareef, Abdi, and Fahad 2020). In this approach, seasonal differences indicated by experiments under controlled conditions (Du, Kruse, et al. 2021) were not considered. At strong heat in summer, foliar water and chlorophyll contents as well as photosynthesis were reduced, but enhanced contents of anthocyanins, carotinoids, H_2_O_2_ and malondialdehyde (MDA) were recorded in the leaves. These responses were maintained when date palms were additionally exposed to drought or salinity in summer climate. Under these conditions, also ABA and proline contents were enhanced. When temperature declined in autumn, foliar water contents increased, photosynthesis recovered and ABA and proline contents decreased, even if drought and salinity were maintained.

## 7 Conclusions and Future Required Research Actions

This review extends our knowledge by establishing that date palms have developed a multitude of interacting common and stress‐specific biochemical defence mechanisms to thrive in desert environments (Table 1). In addition to incorporating avoidance, osmotic and metabolic adjustments as well as ROS scavenging in leaves and roots used by many other plant species, the mechanisms applied by date palm include efficient root‐to‐shoot communication, even when stomatal closure limits xylem transport. Additionally, they emphasize the role of heat shock proteins in temperature stress protection and unique root adaptations that enhance ozone, drought and salinity tolerance. On the other hand, our results show that approaches to enhance stress tolerance of susceptible species that focus on individual stress compensation mechanisms are unlikely to be successful in extreme environments. By demonstrating how these mechanisms work together, the review provides a solid basis for future research aimed at improving crop resilience.

Advanced molecular breeding techniques can expedite the adoption of these traits, creating crops suited for arid regions and increasing productivity in areas affected by climate change. To fortify crops against heat, water scarcity and salinity, a multipronged approach combining strategies like efficient photosynthesis, osmotic adjustments, ion compartmentalization, and antioxidant defences is essential. The ability of halophytes to sequester sodium and accumulate protective solutes suggests that multigene engineering could be more effective than single‐gene modifications. Hormone signalling, root system adjustments, and transcription factor engineering also play pivotal roles. Additionally, harnessing root‐associated microbes may improve nutrient uptake and bolster crop resistance—a hypothesis that awaits clarification. By integrating these strategies, we may cultivate crops better equipped to withstand drought, saline and other extreme environments.

Still the mechanisms that allow date palm to grow and develop in the desert are not fully understood. Therefore, future research actions are required, in particular, research aiming at elucidation of the mechanisms that avoid toxic levels of salts in the cytosol, compensate simultaneous exposure to multiple types of stress, balance the trade‐off between stress compensation/defence and growth, as well as fruit production, and generate necessary energy to support defence processes under stress. In addition, most studies reported focused on stress compensation mechanisms in the leaves and omitted the roots, although they are the first target of several stresses such as drought and salinity. Most importantly, current information is mostly from controlled environment approaches with seedlings/small tress. Knowledge from experimental field studies with mature trees is lacking. Furthermore, to enhance date palm resilience in arid environments, microbial associations may become crucial. Metagenomic analyses can identify beneficial microbes in the rhizosphere and pedosphere. Isolating and studying plant growth‐promoting rhizobacteria and arbuscular mycorrhizal fungi will reveal their roles in nutrient acquisition, stress alleviation, and phytohormone production. Field trials with selected microbial consortia on young date palms will assess their impact on growth, stress tolerance, and yield. Optimized microbial formulations can then be developed for large‐scale application. Integrating microbial traits into breeding programs may aid in selecting resilient date palm varieties. This could be a game‐changer, enhancing stress tolerance in desert flora and other plants under multifaceted challenging conditions.

## Conflicts of Interest

The authors declare no conflicts of interest.

## Data Availability

Data sharing is not applicable to this article because no data sets were generated during the current study.
